# Supplementary material for: Yeast facilitates the multiplication of Drosophila bacterial symbionts but has no effect on the form or parameters of Taylor’s law
Source: PLoS One. 2020 Nov 23;15(11):e0242692. doi: 10.1371/journal.pone.0242692 (PMC7682849; doi:10.1371/journal.pone.0242692)
Supplement: Attachment — Submitted filename: Response to Reviewers.docx [file pone.0242692.s001.docx]

**Response to Reviewers**

Manuscript: Yeast facilitates the multiplication of *Drosophila* bacterial symbionts but has no effect on the form or parameters of Taylor’s law

*Decision:*

*by Guido Favia, 2020-07-09*

Thank you for submitting your manuscript to PLOS ONE. After careful consideration, we feel that it has merit but does not fully meet PLOS ONE’s publication criteria as it currently stands. Therefore, we invite you to submit a revised version of the manuscript that addresses the points raised during the review process.
In particular I invite you to respond to the concerns raised by referee 1.

Dear Editor,

Thank you very much. In the revised manuscript, we address the points raised during the review process. Please find our response to each point below.

*Journal requirements:*

1. Please ensure that your manuscript meets PLOS ONE's style requirements, including those for file naming.

We formatted the revised manuscript according to these documents. In addition, we uploaded our figure files to the PACE digital diagnostic tool and verified that figures meet PLOS requirements.

2. In your Methods section, please provide additional location information of the collection sites, including geographic coordinates for the data set if available.

The biological material was obtained from several private places in and near Montpellier, southern France. We added some location information.

3. In your Methods section, please provide additional information regarding the permits you obtained for the work. Please ensure you have included the full name of the authority that approved the collection sites access and, if no permits were required, a brief statement explaining why.

We added this information.

*Review Comments to the Authors:*

*Reviewer #1*

The manuscript by Guilhot and colleagues is very interesting and proposed a new aspect to look for in the interactions between microorganisms. Although the topic is very interesting, the study presents a few major weaknesses that should be addressed before the manuscript is suitable for publication.

General considerations:

Although the abstract, introduction and material and methods are well written, results should be more detailed while the discussion should be shortened.

Thank you for these positive comments. Following your helpful feedbacks we substantially shortened the discussion. The results section remained however fairly short as it is comprehensive and could not be made longer.

Specific comments:

1. In the experimental design, at the end of the experiment, the authors sampled bacteria from the fruit pulp for cell counts. It is not clear how the authors ensured that the sampled microorganisms are the bacteria that they inoculated at the start of the experiment and not a consortium formed by their bacteria of interest and the endophytes already present in the fruit pulp. If the authors have taken measures to ensure the isolation of only the bacteria that they inoculated it would be important to add this step in the description of the experimental design. If the authors did not implement a protocol to isolate their bacteria nor to ensure that the microorganisms that they isolated are indeed the bacteria that they inoculated, there is a possibility that the bacterial densities that they measure are not those of the bacterial species they inoculated but of that of the cultivable bacterial community that was present in the fruit pulp at the end of the experiment. If this is the case both the results and the discussion should be modified to take into account this possibility.

Referee 1 raises a very important point. We agree entirely about the importance of distinguishing contaminants from inoculated microorganisms. We are certain the bacteria we counted in the fruit flesh at the end of the experiment were the ones we inoculated for two main reasons. Both relate to the fact we were able to recognize most of the strains we used on the basis of colony morphology and metabolic ability (see Table 1 for growth medium and incubation temperatures).

1. Bacteria-free control fruits (i.e. fruits not inoculated with bacteria) remained free of culturable bacteria. It is therefore unlikely bacteria counted in inoculated fruits were contaminants or endophytes.

2. In inoculated fruit, we never observed bacteria with a metabolic profile or a colony morphology different from that of the strain we had inoculated. Even though we can only discriminate about half of our strains with these criteria, it would be very unlikely cross-contaminants or endophytes would have only occurred in treatments that could not be discriminated.

We added these elements of reasoning at the end of ‘Material and methods: Experimental design’ section (lines 117-125).

2. The a and b of Taylor’s power law [log(variance of population density)= a + b log(mean of population density)] are specific to the species but in this study, these two parameters are inferred globally for different species. The authors should recalculate the two parameters singularly for each species and include them in a result table and base their result description and discussion section on that table.

In our study of bacterial densities, we estimate a mixed-species Taylor’s power law. In this common type of Taylor’s power law the a and b parameters are estimated from mean and variance of numbers of different taxa (20 bacteria) computed from simultaneous samples from multiple sites (petri dishes).

A number of examples of mixed-species Taylor’s laws are presented in the book of R.A.J. Taylor (2019) ‘Taylor’s power law: order and pattern in nature’. Academic Press. Here are two examples from this book:

Figure A (p.71): Freshwater plankton mixed-species TPL of experiments conducted in 2004 and 2006 do not differ in slope or intercept. Each point is a species found in samples from a reservoir in Siberia. Circled points are of the noxious cyanobacterium *Anabaena flos-aquae* and the squared outliers are of cyanobacterium *Synechocystis salina*. Data from Table 3 in [Kravchuk et al. (2011) Mar. Freshw. Res.](http://www.publish.csiro.au/mf/MF10256)

Figure B (p.152): Mixed-species ensemble TPL of nematodes (mostly genus, but some species and families) in a seminatural grass pasture in Sweden and labeled by trophic groups are fit by a single regression. Data from Table 2 in [Viketoft (2007) Nematology.](https://brill.com/view/journals/nemy/9/1/article-p131_13.xml)

We modified the ‘Material and methods’ and ‘Results’ parts of our manuscript to identify explicitly what we now call mixed-species Taylor’s law (bacterial strains), and single-species Taylor’s law (yeast strain) (lines 139, 145, 188)

It was not possible to calculate a and b parameters for each bacterial strain separately as we have only 6 or 7 estimates of cell density per bacterial strain (i.e. 6 or 7 replicates) while several means and variances are necessary to test Taylor’s law and estimate parameters.

3. In the figures 1A and 2B the data points represent independent results and should not be linked by a line.

We modified the figures according to this point. Note that the figures concerned by this modification were 1B and 2A and not 1A and 2B.

4. line 223-225: “In our experiment … behavior”, The experimental design is not able to assess the effect on the yeast due to the direct or indirect (bacteria interacting with the insect) interactions with Drosophila especially since both bacteria and yeast are inoculated to the fruit pulp and later isolated from it. In this experiment, Drosophila represents only another variable present in all the experiments. In order to observe a potential effect due to the interaction with Drosophila, the authors should have planned for another series of experiment replicating the entire setup but without adding the fly.

This section was accordingly edited.

5. line 227-237: “Host-mediated … participate in”, since the experimental design does not allow to test the effects of the microorganisms on the fly nor does it allow to assess the effects of the insect on the microorganisms, this part should be removed since it is highly speculative in regards to the experiment.

This section was accordingly edited.

6. line 258-259: “In our experiment … since 2^10 = 1000.”, The inference that the authors had 10 generations based on the final number of bacteria will hold only if the bacteria are in an exponential phase of growth which is highly improbable after 7 days in a closed system (the petri dish). Some of the bacteria used in the experiment are known to have a short generation time and will probably reach their highest density in a short amount of time, after which their apparent growth will halt (plateau phase). In this phase, the number of new bacteria with bacteria multiplying but generations appearing but the total number of the population is constant because of the dying bacteria. Because of the dynamics of bacterial populations in a closed system, unless the bacteria are in an exponential growth phase it is very hard to determine the number of generations that have passed based on the final population size.

Thank you for this very insightful comment! We modified the discussion accordingly.

*Reviewer #2*

The article gives an insight of how the interactions between microbial symbionts may influence theirs and host's demography. The results show new prospectives on the relationship between Drosophila melanogaster and yeast and bacteria.

**

Thank you for the encouraging comments.

In addition, we declare that we slightly modified one of our statistical analyses. To test whether numbers of yeast and bacterial cells correlated among bacterial treatments in our experiment, we used a Reduced Major Axis regression in the previous version of the manuscript. In the present version of the manuscript, we used a Major Axis regression, which is more adapted to our data. This new regression method changed some of our results (lines 171:178). We modified the ‘Material and methods’ and ‘Results’ parts of our manuscript as well as Figure 2B accordingly.

The authors.
